# Subchronic Peripheral Neuregulin-1 Increases Ventral Hippocampal Neurogenesis and Induces Antidepressant-Like Effects

**DOI:** 10.1371/journal.pone.0026610

**Published:** 2011-10-19

**Authors:** Ian Mahar, Stephanie Tan, Maria Antonietta Davoli, Sergio Dominguez-Lopez, Calvin Qiang, Adeline Rachalski, Gustavo Turecki, Naguib Mechawar

**Affiliations:** 1 McGill Group for Suicide Studies, Douglas Mental Health University Institute, McGill University, Verdun, Québec, Canada; 2 Department of Psychiatry and McGill University, Montréal, Québec, Canada; 3 Department of Neurology and Neurosurgery, McGill University, Montréal, Québec, Canada; University of Nebraska Medical Center, United States of America

## Abstract

**Background:**

Adult hippocampal neurogenesis has been implicated in the mechanism of antidepressant action, and neurotrophic factors can mediate the neurogenic changes underlying these effects. The neurotrophic factor neuregulin-1 (NRG1) is involved in many aspects of brain development, from cell fate determination to neuronal maturation. However, nothing is known about the influence of NRG1 on neurodevelopmental processes occurring in the mature hippocampus.

**Methods:**

Adult male mice were given subcutaneous NRG1 or saline to assess dentate gyrus proliferation and neurogenesis, as well as cell fate determination. Mice also underwent behavioral testing. Expression of ErbB3 and ErbB4 NRG1 receptors in newborn dentate gyrus cells was assessed at various time points between birth and maturity. The phenotype of ErbB-expressing progenitor cells was also characterized with cell type-specific markers.

**Results:**

The current study shows that subchronic peripheral NRG1β administration selectively increased cell proliferation (by 71%) and neurogenesis (by 50%) in the caudal dentate gyrus within the ventral hippocampus. This pro-proliferative effect did not alter neuronal fate, and may have been mediated by ErbB3 receptors, which were expressed by newborn dentate gyrus cells from cell division to maturity and colocalized with SOX2 in the subgranular zone. Furthermore, four weeks after cessation of subchronic treatment, animals displayed robust antidepressant-like behavior in the absence of changes in locomotor activity, whereas acute treatment did not produce antidepressant effects.

**Conclusions:**

These results show that neuregulin-1β has pro-proliferative, neurogenic and antidepressant properties, further highlight the importance of peripheral neurotrophic factors in neurogenesis and mood, and support the role of hippocampal neurogenesis in mediating antidepressant effects.

## Introduction

Adult hippocampal neurogenesis, the evolutionarily conserved process by which new granule cell neurons are added to the dentate gyrus (DG) of the hippocampus, has been implicated in several brain functions, including affective modulation. According to the neurogenic theory of antidepressant effects, increasing hippocampal neurogenesis can ameliorate depressive symptoms ([1]–[8]; see [9]–[11] for review), although it is controversial whether deficits in neurogenesis are sufficient to cause a depressive phenotype [5], [12]–[16]. In support of the involvement of neurogenesis in antidepressant effects, antidepressant treatments increase hippocampal neurogenesis [1], [17], ablating hippocampal neurogenesis can prevent antidepressant effects ([3], [5], [12], [18]; although see [13], [19]), and the latency of these effects in humans and animals correlates with the time required for newborn DG neurons to integrate into mature circuits [6].

Neurotrophic factors have been proposed to mediate the neurogenic response to antidepressants, with strong evidence that peripherally circulating neurotrophic factors can modulate both mood and neurogenesis [20]–[25]. In particular, chronic peripheral administration of brain-derived neurotrophic factor (BDNF) has been reported to increase neurogenesis [25] and to induce antidepressant and anxiolytic effects [25], [26]. Interestingly, serum levels of BDNF are also reduced in depressed patients and increased by antidepressant treatment [23], [27], raising the possibility that peripheral activity of this neurotrophic factor could affect mood by modulating central neurophysiological processes such as adult hippocampal neurogenesis. Further support for a peripheral influence of neurotrophins on mood arises from the demonstration that peripheral administration of the pro-neurogenic hormone insulin-like growth factor-I, which can cross the blood-brain barrier (BBB), produces antidepressant-like behavior [21].

The neuregulin (NRG) family of epidermal growth factor-related proteins comprises a wide variety of soluble and membrane-bound proteins that mediate their effects through ErbB2-ErbB4 tyrosine kinase receptors [28], [29]. The diversity of NRG proteins, arising mainly from alternative splicing, is particularly well-documented for the neurotrophic factor NRG1, which includes at least fifteen isoforms. In addition to being expressed in the brain, some NRG1 isoforms are also widely expressed in the periphery [30]–[32]. In particular, NRG1β, the most widespread NRG1 isoform in the brain, is found in the circulation, from which it can readily cross the adult BBB via carrier-mediated transport and affect brain activity [33]–[36]. Studies of NRG1 signaling in the brain indicate prominent roles for this protein during development, such as cell fate determination [37], axon guidance [38], radial glia elongation [39]–[41], neuronal migration [42], [43] and dendritic growth [44].

Despite our rapidly expanding knowledge of NRG1 functions in the developing brain, very little is currently known about the influence of this neurotrophic factor on neurodevelopmental processes occurring at maturity. NRG1 administration in the adult subventricular zone (SVZ), the main proliferative region in the adult brain, has been shown to affect progenitor organization and migration [45]. However, a possible role for NRG1 signaling in modulating hippocampal neurogenesis remains to be examined [46]. Here, we exploited the fact that NRG1β readily crosses the BBB to study the consequences of subchronic peripheral administration of this neurotrophic factor on hippocampal neurogenesis. Based on previous studies showing that NRG1 is pro-proliferative in vitro[47], [48] and that neurotrophic factors increase proliferation and neurogenesis in the adult hippocampus [20], [22], [25], we hypothesized that NRG1β treatment would stimulate DG proliferation, leading to an increase in hippocampal neurogenesis. We further hypothesized that this increase in neurogenesis would lead to antidepressant effects, in line with studies demonstrating an association between these phenomena [3], [5], [12], [18], as well as those showing antidepressant effects after neurotrophic factor administration [21], [25]. We show that this treatment strongly stimulates DG cell proliferation exclusively in the ventral hippocampus, leading to an increase in neurogenesis that is accompanied by robust antidepressant-like behavior.

## Materials and Methods

### Animals

Adult male C57BL/6 mice were purchased from Charles River Canada. For ErbB3/SOX2/nestin triple labeling, group-housed transgenic mice expressing GFP at the nestin promoter (nestin-GFP; [49]) on a C57BL/6 background were used. All animals were 2 months of age and housed in groups (except animals for neurogenesis and behavioral experiments, which were isolated) on a 12∶12 light:dark cycle with *ad libitum* access to food and water. All experiments followed the policies and guidelines of the Canadian Council on Animal Care and were approved by McGill University's Animal Care Committee (approval ID: 5473).

### NRG1β and BrdU administration

After anesthesia with isoflurane, mice were implanted with subcutaneous osmotic mini-pumps (Alzet) containing either recombinant NRG1β type-I (EGF domain dissolved in sterile 0.9% saline, administered at a constant rate of 10 µg/d; R&D Systems; accession # NP_039250) or vehicle (randomized groups assignment). Animals were given a subcutaneous saline injection and placed on a heating pad to recover, and were monitored post-operatively for complications. An anti-inflammatory Carprofen tablet was placed in each cage. Depleted mini-pumps were removed from anesthetized animals after the administration period, and pump depletion was verified.

**Figure 1:**
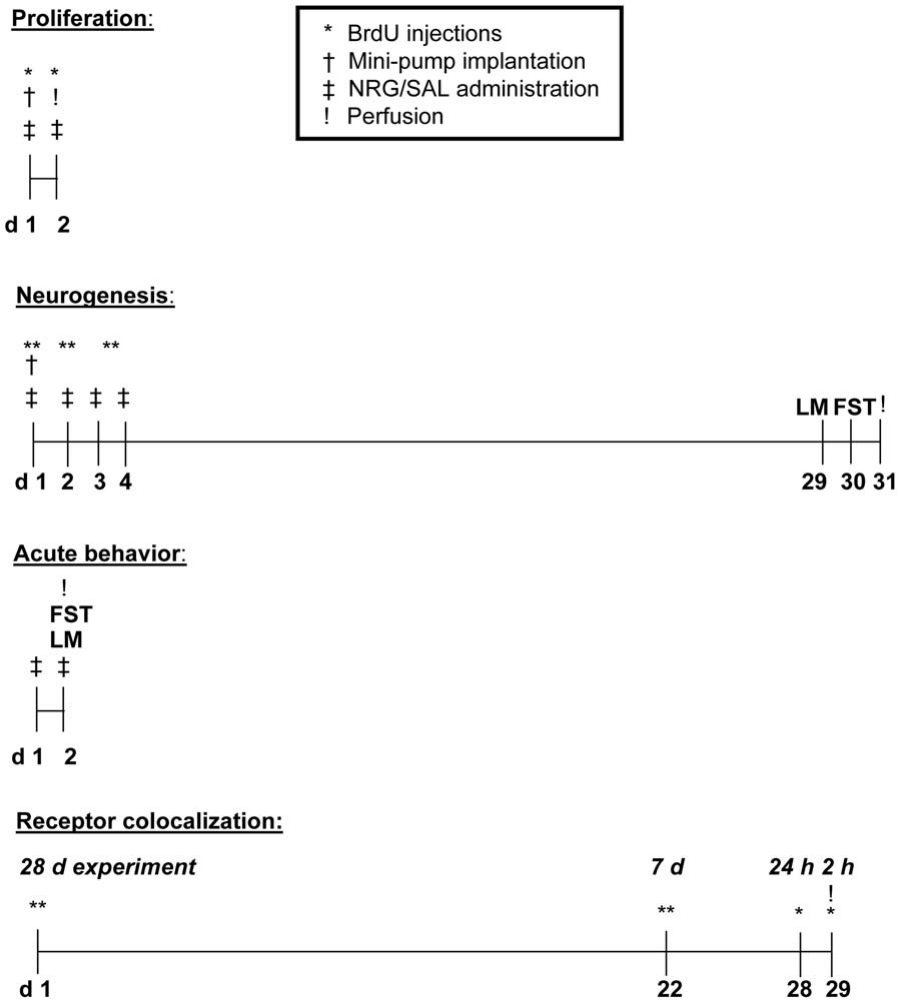
Experimental timelines. LM, locomotor task; FST, forced swim test.

To evaluate proliferation, mice (n = 6/group) were sacrificed at the end of a 24 h NRG1β or vehicle administration period, having received two injections of bromodeoxyuridine (BrdU; Sigma; 50 mg/kg, i.p.) dissolved in sterile 0.9% saline with 0.4 M NaOH: one immediately after mini-pump implantation, and the other two hours prior to sacrifice. To evaluate neurogenesis (encompassing proliferation, differentiation and survival), mice (n = 7/group) received BrdU twice daily during the 72 h NRG1β or vehicle administration period and sacrificed 30 d after mini-pump implantation. For immunohistochemical (IHC) receptor colocalization experiments, naive animals received BrdU 2 h, 24 h, 7 d, or 28 d prior to sacrifice (n = 2/survival period), with the latter two groups receiving two BrdU injections on the day of administration. To determine the acute behavioral effects of NRG1β, mice were given three 0.1 ml i.p. injections of NRG1β (3.33 µg; n = 7) or vehicle (n = 8), 24 h, 12 h and 1 h prior to testing, and then sacrificed (see **Fig. 1** for experimental timelines).

### Tissue processing

Animals were deeply anesthetized with a cocktail of ketamine, xylazine and acepromazine (0.1 ml/100 g), and perfused through the heart with ice-cold phosphate-buffered saline (PBS) followed by 4% formaldehyde in 0.1 M phosphate buffer. Brains were then rapidly removed, postfixed at 4°C for 24 h in fixative, then transferred to a 30% sucrose solution until equilibrium was reached. Brains were cut using a cryostat into serial 40 µm-thick coronal sections, which were placed in a cryoprotectant solution (glycerol:ethylene glycol:PBS, 3∶3∶4) and stored at −20°C.

### BrdU immunohistochemistry

Unless otherwise specified, all IHC incubations were at room temperature. The section-sampling fraction was 1/8. Omitting primary antibodies resulted in an absence of specific staining for all IHC protocols. Rinses with PBS preceded all steps except the addition of primary antibodies and addition of H_2_O_2_. For BrdU light microscopy IHC, sections were pre-treated for 1.5 h in PBS containing 0.2% Triton X-100 (PBS-T; Fisher), 10 min in PBS containing 0.9% H_2_O_2_, and 30 min at 37°C in PBS with 2N HCl to denature DNA. Sections were then incubated in PBS-T containing 2% Normal Goat Serum (NGS) for 30 min, and then overnight at 4°C in the same solution with monoclonal rat anti-BrdU (1∶1000; Serotec). Sections were then incubated for 1 h in biotinylated goat anti-rat antibody (1∶200; Vector Laboratories), followed for 30 min by the avidin-biotin complex procedure (ABC Kit, Vectastain Elite, Vector). Labeling was revealed with a diaminobenzidine kit (Vector). Sections were mounted on glass slides, dehydrated, and coverslipped with Permount (Fisher Scientific).

### BrdU/NeuN double-labeling

To determine the proportions of newborn cells that became neurons, coronal brain sections from the neurogenesis experiment were incubated overnight at 4°C with rat anti-BrdU (as above) and mouse anti-NeuN (1∶200; Chemicon) antibodies in 2% NGS in PBS-T, after consecutive incubations in PBS-T (2 h), 2N HCl in PBS at 37°C (30 min) and PBS-T containing 2% NGS (1 h). This was followed by a 1.5 h incubation in fluorescent DyLight 594-labeled goat anti-rat (1∶1500; Jackson) and DyLight 488-labeled goat anti-mouse (1∶500; Jackson) antibodies in 2% NGS in PBS-T. Sections were mounted on glass slides and coverslipped with ProLong Gold antifade reagent with DAPI (Invitrogen).

### BrdU/ErbB double-labeling

Sections from animals injected with BrdU 2 h, 24 h, 7 d, or 28 d prior to sacrifice were incubated for 2 h in PBS-T. Sections were then incubated for 30 min in PBS at 37°C in 1N HCl to denature DNA, and incubated in 2% NGS in PBS-T for 2 h before an overnight incubation at 4°C in rat anti-BrdU (as above) and monoclonal mouse anti-ErbB4 (Thermo Scientific; 1∶100) or monoclonal rabbit anti-ErbB3 (accession # Q61526, Santa Cruz; 1∶100) in the same solution. Sections were then incubated for 1.5 h in DyLight 594 goat anti-rat (1∶1500) and DyLight 488 goat anti-rabbit (1∶500) or goat anti-mouse (1∶100 or 1∶200) antibodies (Jackson) in PBS-T containing 2% NGS. Sections were mounted on slides and coverslipped and coded as above.

The anti-ErbB4 antibody is directed against an extracellular domain of the ErbB4 receptor [50], and the anti-ErbB3 antibody is directed against the C-terminal domain [51]. The specificity of the latter antibody has been validated previously in ErbB3-knockout tissues [52], [53]. In addition, we confirmed the staining pattern using a different anti-ErbB3 antibody raised in mice (Millipore, 1∶25, 1∶50 and 1∶100; accession # P21860; not shown) under light microscopy conditions with DAB. Furthermore, immunofluorescence double-labeling with both anti-ErbB3 antibodies revealed colocalization throughout the DG (wide-field and confocal microscopy).

### ErbB3/SOX2/nestin triple-labeling

Nestin-GFP mouse brain sections were incubated for 2 h in PBS-T and 1 h in 2% NGS in PBS-T, then overnight at 4°C in rabbit anti-ErbB3 (as above) and mouse anti-SOX2 (R&D 1∶200; accession # P48431). The staining pattern of the latter (which has been validated previously in SOX2-knockout tissue [54]) was confirmed with an additional anti-SOX2 antibody (Millipore; 1∶12 000 and 1∶20 000; accession # P48431; not shown) under light microscopy conditions with DAB. Secondary antibodies (DyLight 594 goat anti-rabbit and Dylight 649 goat anti-mouse, 1∶500, Jackson) were added for 1.5 h. Sections were mounted on slides and coverslipped as above. As a negative control, a nestin-GFP-negative sibling did not show GFP staining using wide-field and confocal microscopy.

### Cell quantification

All quantifications and analyses were done by an investigator blind to experimental groups. BrdU-immunoreactive (-IR) cells in the DG of the hippocampus were counted using a Leica CME with a 40X 0.65NA E2 achromat objective (proliferation) or a Leica DM 2500 with a 40X 0.75 NA HCX PL Fluotar objective (neurogenesis). Cell counts were expressed as average numbers per DG. Proliferation of cells in the SVZ was determined at 40X (0.75 NA UPlan FL N objective) on an Olympus BX51 microscope equipped with a motorized stage and CX-9000 camera (MBF), using an optical fractionator (OF) probe (Stereo Investigator, MBF). A pilot study conducted to establish sampling parameters revealed a Gunderson coefficient of error (CE) (m = 1) <0.05. The size of the counting frame for cell estimation was 500 µm^2^, dissector height was set to 14 µm, and guard zone distance to 1 µm. The average sampling area was 6072.5 µm^2^. As the OF procedure provided cell count estimates and not absolute cell numbers, density of BrdU-IR cells was determined as the estimated population using section thickness divided by the Cavalieri volume corrected for overprojection. Gunderson CEs (m = 1) for all animals were <0.05.

### Surface area and volumetric analyses

To rule out group differences in surface area or volume (as calculated by planimetry and Cavalieri estimation), the SVZs and DGs of each brain used in cell quantification were traced on the Olympus BX51 microscope mentioned above with a 10X UPlan FL N 0.3 NA objective using the Stereo Investigator software package (MBF). SVZs and DGs as well as rostral (−1.46 to −2.54 mm from bregma [55]) and caudal (−2.55 to −3.80 mm from bregma) DG subregions presented no significant difference between groups for any of the measures of this analysis, including total and average surface area and total and average volume, both corrected and uncorrected for overprojection (ps>0.05).

### Confocal microscopy

Multiple-labeling analyses were conducted at 40X (1.3 NA Plan-Neofluar 40x Oil DIC objective) on a Zeiss LSM510 Meta confocal microscope equipped with an Axiovert 200 M stand and motorized stage (Carl Zeiss Canada), using 405 nm, 488 nm, 543 nm, and 633 nm wavelength lasers. Images were obtained using the Zeiss Aim software package (Carl Zeiss Canada), at a pixel size of 0.11 µm for x and y, a scan average of ≥4 frames, a pixel dwell time of ≥3.20 µs, and optical slice of <3 µm sampled at an interval of <1.5 µm. Images were unaltered except for overall brightness and contrast. For quantitative colocalization analyses, 20 random cells from the rostral and caudal DG (10/subregion from multiple DGs) of each animal were analyzed, and data expressed as the percentage of BrdU-IR cells that were also NeuN-IR or ErbB-IR.

### Behavior

Animals in the neurogenesis experiment were assessed in a locomotor task and in the forced swim test (FST) 28 d and 29 d, respectively, after the onset of the 72 h NRG1β or saline administration period, or 40 min and 1 h after the final injection in the acute behavior experiment. The forced swim test (FST) was chosen for its implication as a hippocampal neurogenesis-dependent task ([12], [18]; although see [13], [19]), and the locomotor task served to determine whether putative antidepressant effects were due to changes in general locomotor activity. To study locomotor behavior, mice were placed individually into acrylic Versamax RS2USB v4.00 Animal Activity Monitor boxes (.2 m L×.2 m W×.3 m H; Accuscan) in a room lit by red light for a period of 90 min (20 min for the acute behavior experiment). Locomotor behavior was analyzed automatically by the Versamax VMX 1.4B software system (AccuScan). Three periods of activity were analyzed: no habituation (first 10 min activity), 10 min of habituation (second 10 min activity) and extended habituation for the neurogenesis experiment (30 min habituation, 60 min activity). For the FST, mice were placed in 4 L glass beakers (25 cm depth, 15 cm diameter) 1/3^rd^ filled with water (25°C) for a period of 10 minutes in a dimly lit room. Behavior was recorded by video equipment and subsequently analyzed using Videotrack behavioral tracking software (Viewpoint). The first two minutes were treated as habituation, and the following four minutes analyzed for duration of swimming and immobility behavior.

### Statistics

Normality of data was assessed using Shapiro-Wilk tests. Parametric pair-wise comparisons were made using Student's t-tests. Non-parametric pair-wise comparisons were made with Mann-Whitney U-tests. Comparisons within testing period time points across the duration of the FST were made with Bonferroni-corrected t- and U-tests. Values of p <0.05 were considered to be statistically significant.

## Results

### Proliferation

**Figure 2:**
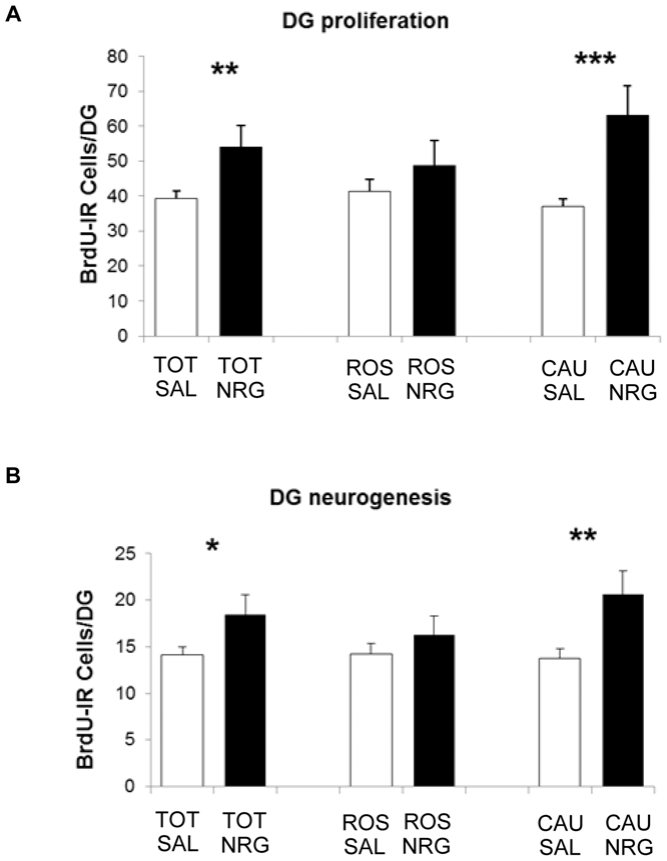
Proliferative and neurogenic effects of peripheral NRG1 administration. (**A**) NRG1 increases DG cell proliferation, in the caudal (71%; p = 0.0011), but not rostral (p = 0.20), DG. (**B**) NRG1 increased the number of BrdU-IR cells 28 d after administration. As with proliferation, this increase is significant in the caudal (50%; p = 0.013), but not rostral (p = 0.29), DG. Bars represent mean ± SEM. *p<0.05; **p<0.025; ***p<0.005. CAU, caudal; NRG, neuregulin; ROS, rostral; SAL, saline; TOT, total.

**Figure 3:**
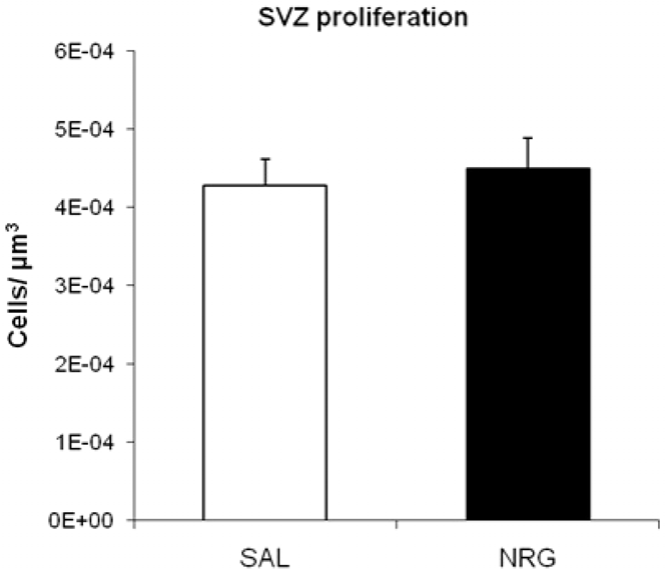
SVZ cell proliferation. NRG1 administration does not affect SVZ proliferation (p = 0.34). Bars represent mean ± SEM. NRG, neuregulin; SAL, saline.

Mice were administered NRG1β or saline for 24 h (to evaluate proliferation immediately after) or 72 h (to evaluate neurogenesis and behavior four weeks later), and received injections of BrdU during administration (see **Fig. 1**). After 24 h of NRG1 administration, cell proliferation in the DG, as assessed by numbers of BrdU–IR cells, increased by 38% compared to controls (p = 0.013). Subregional analyses revealed that this increase was highly significant in the caudal DG (71%; p = 0.0011), but non-significant in the rostral DG (p = 0.29) (**Fig. 2A**). Proliferation in the SVZ did not differ between groups (p = 0.34; **Fig. 3**).

### Neurogenesis

**Figure 4:**
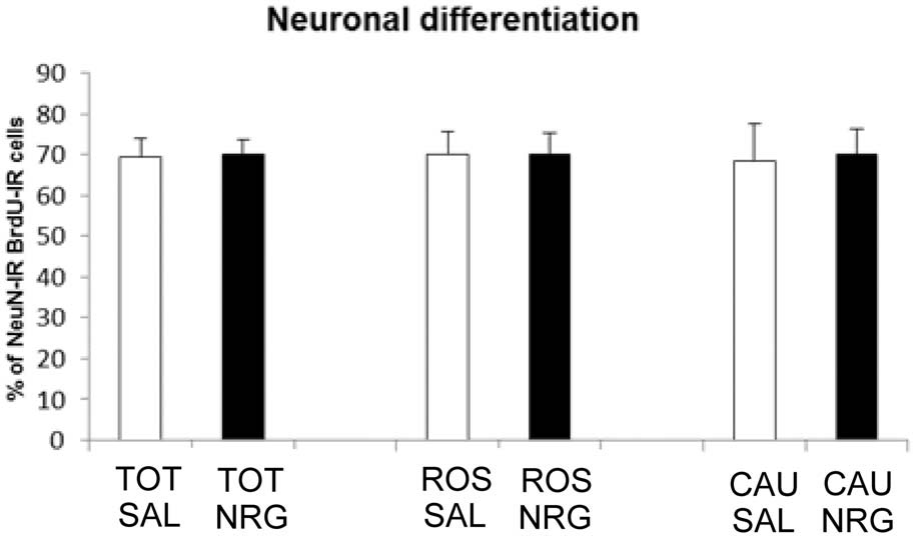
Neuronal differentiation. NRG1 administration does not affect the proportion of BrdU-IR cells that differentiated into NeuN-IR neurons, either overall (p = 0.91) or in the rostral (p = 1.0) or caudal (p = 0.90) subregions. Bars represent mean ± SEM. NRG, neuregulin; SAL, saline.

Overall, BrdU-IR cell numbers in the DG increased four weeks after NRG1β administration (31%; p = 0.042). As with proliferation however, this increase was only significant in the caudal DG (50%, p = 0.013; rostral: p = 0.20) (**Fig. 2B**). The proportion of cells that differentiated into neurons in these animals did not vary with treatment (overall: p = 0.91; rostral DG: p = 1.00 or caudal DG: p = 0.90; **Fig. 4**), indicating that the NRG1β-induced increase in proliferation within the caudal DG led to increased neurogenesis in this region.

### BrdU-ErbB colocalization

**Figure 5:**
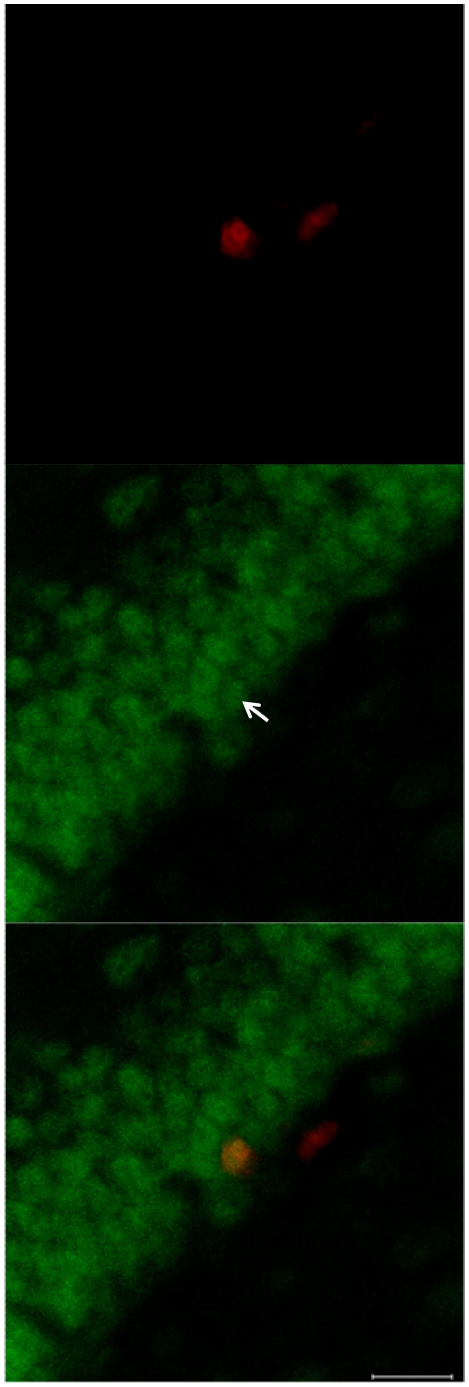
BrdU/ErbB3 colocalization. BrdU-IR cells (**red**) express ErbB3 (**green**), at 2 h (**Figs. S1** and **S2**) and 24 h (**above**), corresponding to the period in which cell proliferation is increased following NRG1 administration, as well as 7 d and 28 d after birth (**Fig. S1 and S2**). Scale bar = 20 µm.

To examine if these effects may have resulted from direct stimulation of NRG1 receptors expressed by progenitor cells, we determined at different post-BrdU injection time points whether BrdU-IR in the DG colocalized with ErbB3- or ErbB4-IR (see **Fig. 1**), as presence of one of these receptors is required for NRG1-ErbB signaling. IR for both receptors were observed in the DG in expression patterns similar to previous studies using these and other antibodies [50], [51], [56]. In naive animals 70±8% (rostral: 55±16%; caudal: 85±3%; mean %±SEM) of newborn cells were found to express ErbB3 during the proliferative period (2 h and 24 h following BrdU injection; **Figs. 5**, **S1** and **S2**). Interestingly, colocalization was also observed 7 d and 28 d post-injection (**Fig. S1** and **S2**), suggesting a life-long influence for NRGs on the activity of adult-born granule cells. BrdU-IR and ErbB4-IR were never found to be colocalized in the DG.

### ErbB3/nestin/SOX2 colocalization

**Figure 6:**
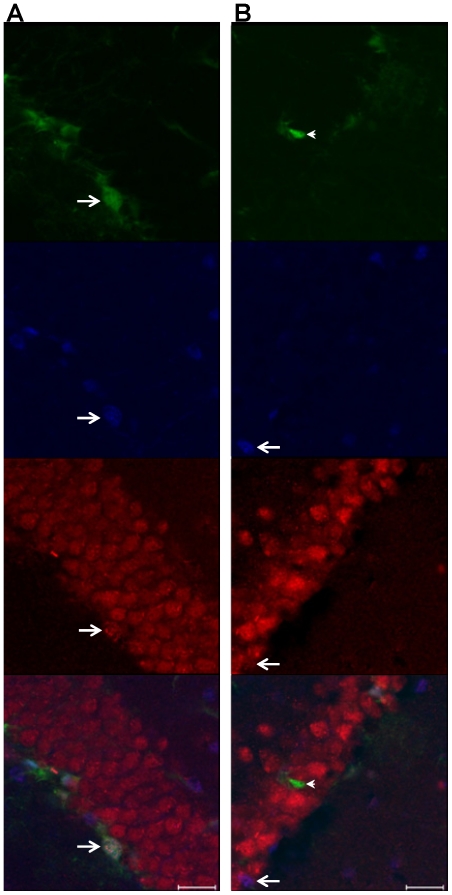
ErbB3/nestin/SOX2 colocalization. ErbB3-IR cells (**red**) in the DG colocalize with nestin (**green**) and SOX2 (**blue**) (arrow in **A**; **Fig. S3A**), or with SOX2 in the absence of nestin (arrow in **B**; **Fig. S3B**), but not nestin in the absence of SOX2. Arrowhead in **B** denotes a nestin-IR cell that is negative for both ErbB3 and SOX2. Scale bar = 20 µm.

To examine which neurogenic cell subtypes in the DG expressed ErbB3, we analyzed the colocalization of ErbB3, nestin and SOX2 in nestin-GFP animals (**Fig 6**). Colocalization was predominately limited to the subgranular zone. Although both SOX2 and nestin colocalized with ErbB3 in the rostral and caudal DG, nestin and ErbB3 were only co-expressed in cells expressing SOX2 (i.e. triple-labeled cells; **Figs. 6A** **and** **S3A**) and not in the absence of SOX2, whereas ErbB3 and SOX2 also colocalized in the absence of nestin (**Fig. 6B** **and** **S3B**), suggesting that the neuronal precursor cells involved in the NRG1-induced proliferative increase express SOX2.

### Behavior

**Figure 7:**
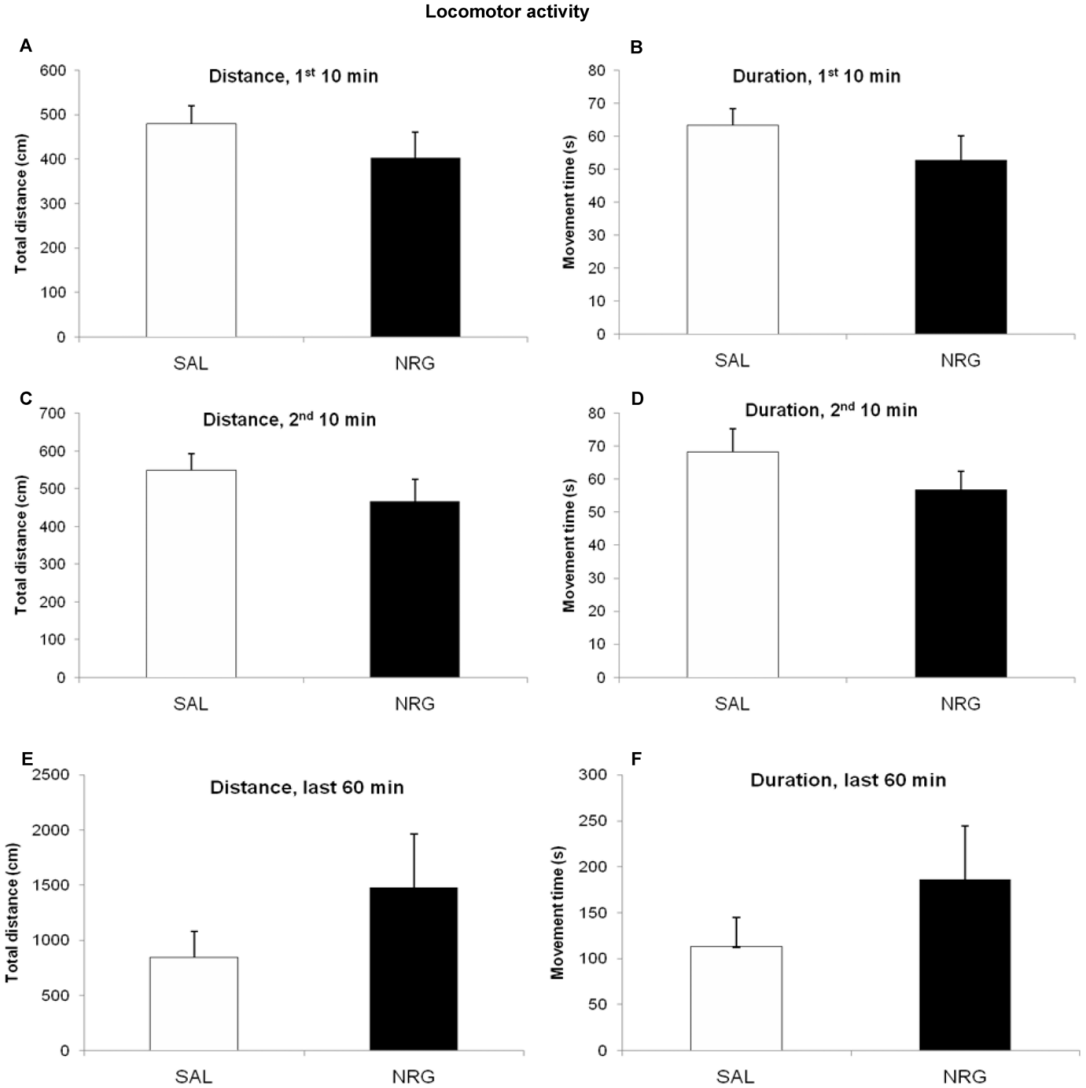
Locomotor activity does not change in response to NRG1 administration. NRG1 administration does not affect locomotor activity 28 d after subchronic administration, either in distance (**A**, **C**, **E**) or duration (**B**, **D**, **F**). **A**, **B**: First 10 min of activity (distance: p = 0.30; duration: p = 0.25). **C**, **D**: 10 min of activity after 10 min habituation (distance: p = 0.28; duration: p = 0.23). **E**, **F**: 60 min of activity after 30 min habituation (distance: p = 0.26; duration: p = 0.29). Bars represent mean ± SEM. NRG, neuregulin; SAL, saline.

**Figure 8:**
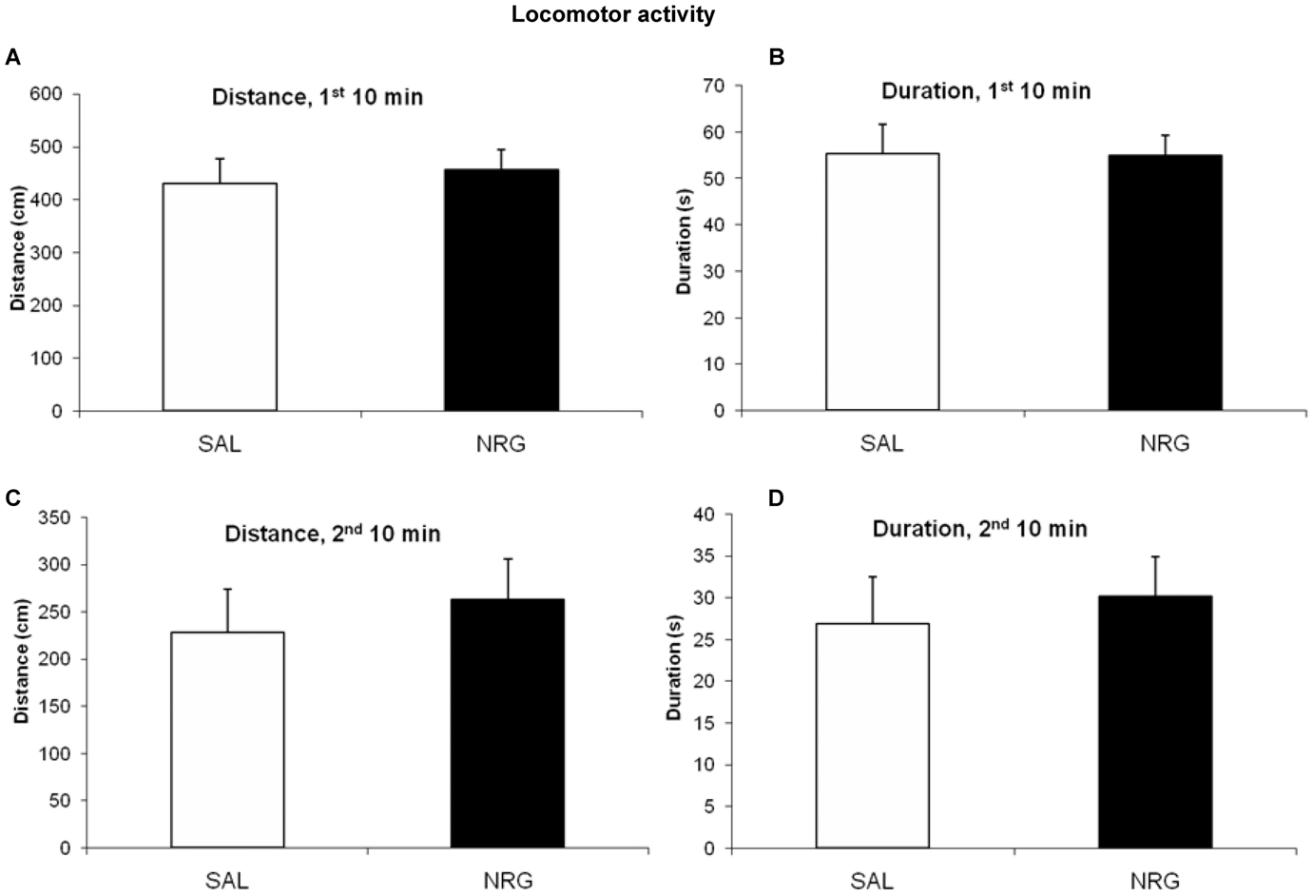
Acute locomotor activity does not change in response to NRG1 administration. NRG1 administration does not affect locomotor activity acutely after administration, either in distance (**A**, **C**) or duration (**B**, **D**). **A**, **B**: First 10 min of activity (distance: p = 0.67; duration: p = 0.96). **C**, **D**: 10 min of activity after 10 min habituation (distance: p = 0.60; duration: p = 0.67). Bars represent mean ± SEM. NRG, neuregulin; SAL, saline.

The effects of subchronic (72 h) NRG1β treatment on antidepressant-like behavior were assessed in the same animals in which neurogenesis was examined. For all analyses of the locomotor task, experimental groups did not differ in either total distance traveled (1^st^ 10 min: p = 0.30; 2^nd^ 10 min: p = 0.28; 60 min after 30 min habituation: p = 0.26) or activity duration (1^st^ 10 min: p = 0.25; 2^nd^ 10 min: p = 0.23; 60 min after 30 min habituation: p = 0.29) (**Fig. 7**). Treatment groups did not differ in locomotor activity after acute treatment (24 h), either initially (distance: p = 0.67; duration: p = 0.96) or after 10 min of habituation (distance: p = 0.60; duration: p = 0.67) (**Fig. 8**).

**Figure 9:**
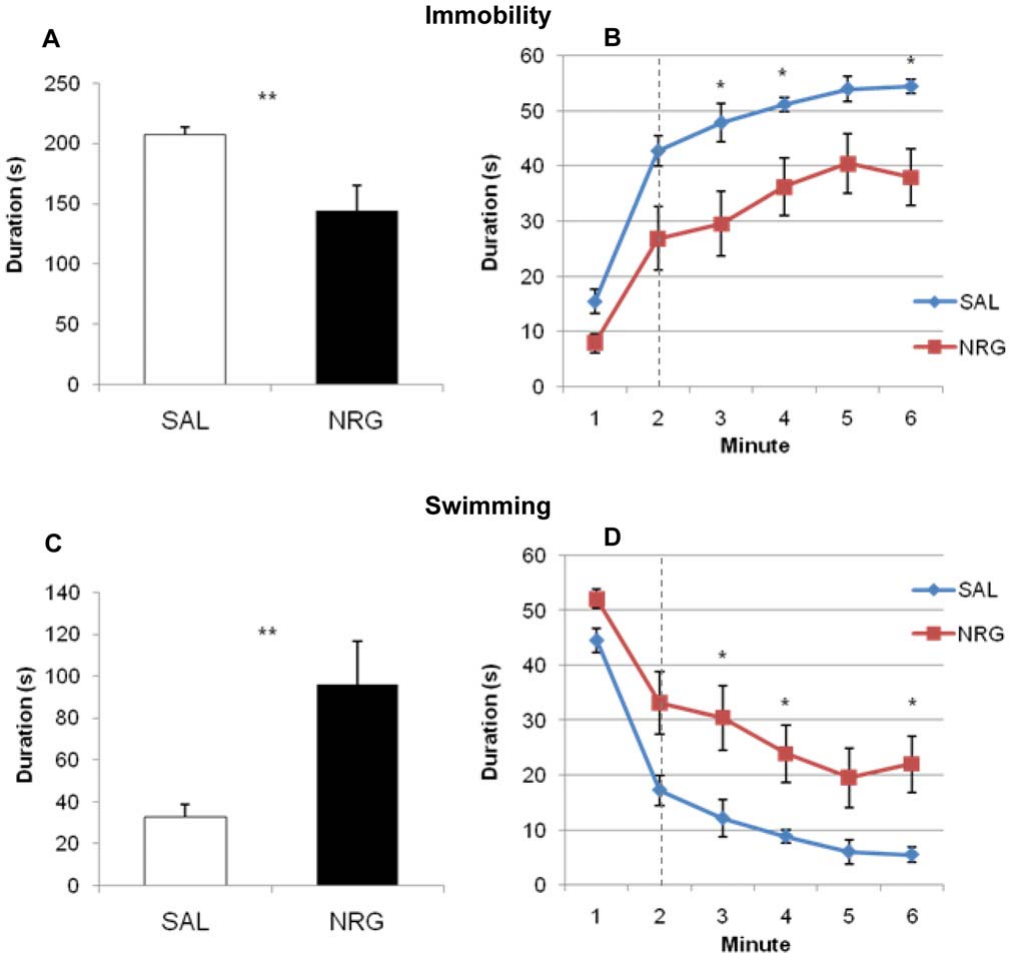
Animals treated with NRG display antidepressant behavior in a forced swim test 28 d after administration. NRG-treated animals show decreased immobility (**A**, 44%, p = 0.0062; **B**) and increased swimming behavior (**C**, 194%, p = 0.0062; **D**), both overall during the testing phase (**A**, **C**) and throughout the task (**B**, **D**). Dashed lines denote beginning of testing phase. Bars and points represent means ± SEM. *p<0.05, **p<0.01. NRG, neuregulin; SAL, saline.

**Figure 10:**
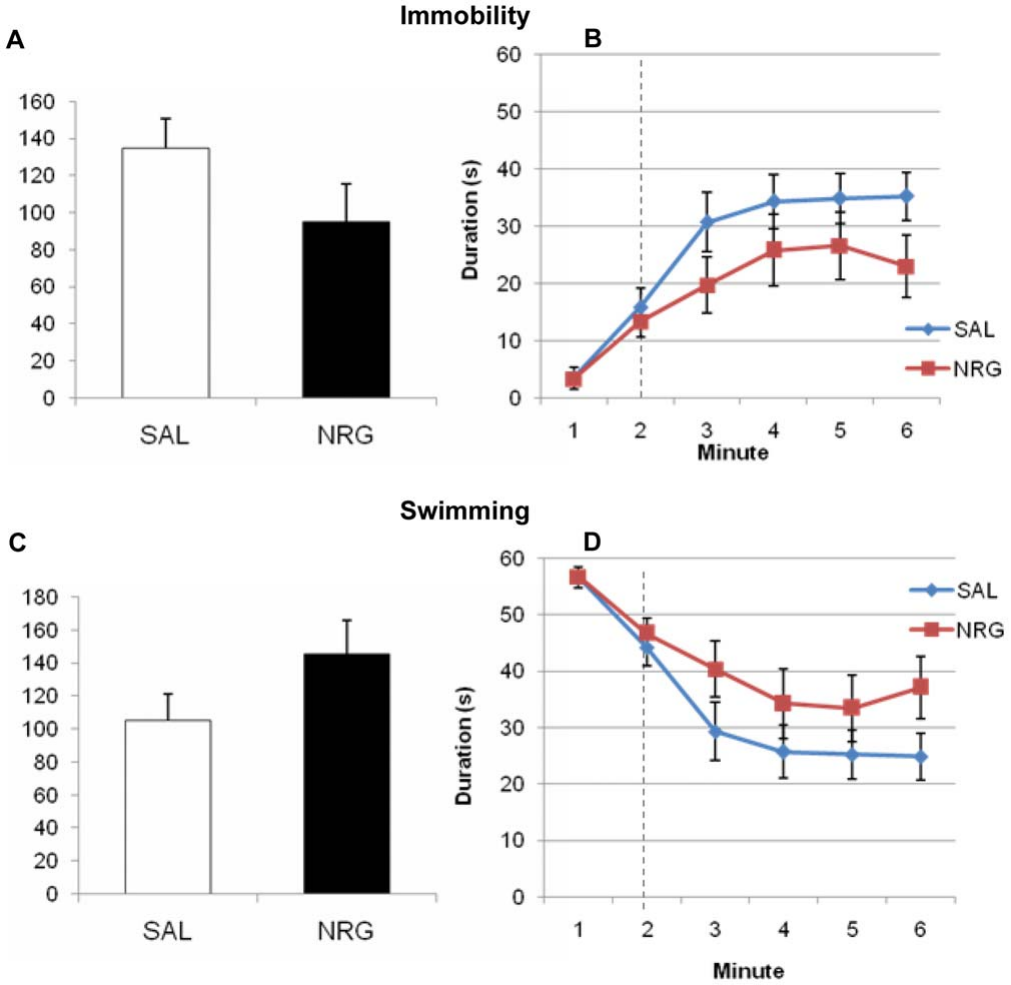
Animals treated with NRG do not display antidepressant behavior in a forced swim test acutely after administration. Treatment groups did not differ with respect to immobility **(A**, p = 0.15; **B**) or swimming behavior (**C**, p = 0.15; **D**), both overall during the testing phase (**A**,**C**) and throughout the task (**B**,**D**). Dashed lines denote beginning of testing phase. Bars and points represent means ± SEM. NRG, neuregulin; SAL, saline.

NRG1β-treated animals showed decreased immobility (44%; p = 0.0062) and increased swimming (194%; p = 0.0062) in the FST four weeks after the administration period, suggesting that NRG1β treatment had antidepressant effects. Bonferroni-corrected temporal analyses revealed that these animals had significantly increased swimming and decreased immobility throughout the testing period (0.019≤ps≤0.039) except the fifth minute (ps = 0.11) (**Fig. 9**). Acute treatment with NRG1β did not produce antidepressant effects in the FST compared to vehicle-treated animals (immobility: p = 0.15; swimming, p = 0.15). During the testing period treatment groups did not differ at any point (0.37≤ps≤1.0) (**Fig. 10**).

## Discussion

This study shows that peripheral NRG1β administration increases cell proliferation in the caudal (but not rostral) DG within 24 h, and that this treatment leads to a similar increase in local neurogenesis. This pro-proliferative effect did not influence cell fate, and was likely mediated by ErbB3 receptors in SOX2-expressing neuronal precursor cells. Given the magnitude of this effect within such a relatively short period, it is likely that the progenitor cells involved in this increase are the rapidly proliferating type-II cells as opposed to the more quiescent type-I stem cells [57], although the widespread expression of ErbB3 in the DG leaves open the possibility of mature granule cells contributing to this phenomenon. In addition, the large increase in DG cell numbers was accompanied by antidepressant-like behaviors four weeks after cessation of subchronic treatment, but not acutely. NRG1β administration did not increase proliferation in the SVZ, in agreement with previous findings [45], suggesting that the pro-proliferative influence of NRG1β in neurogenic regions of the adult brain *in vivo* is restricted to the caudal DG. This region is localized within the ventral hippocampus [17], [58], [59], which is structurally and functionally distinct from the dorsal hippocampus [59]–[61]. Adult ventral hippocampal lesions are anxiolytic and disrupt fear conditioning and memory [60]–[64], whereas neonatal ventral hippocampus lesions are a well-characterized model of schizophrenia [65], [66]. In addition to its role in anxiety, the ventral hippocampus and ventral hippocampal neurogenesis have been implicated in depression, antidepressant-related behavior and response to stress [4], [9], [12], [17], [67]–[72]. The involvement of the ventral hippocampus in emotional modulation may be linked to its connectivity, which includes projections to the prefrontal cortex, amygdala, nucleus accumbens, olfactory bulb, and hypothalamic-pituitary-adrenal axis-related structures [60], [61], [73]–[75], and to its increased serotonergic innervation in comparison with the dorsal hippocampus [9], [76]–[78]. Interestingly, NRG1 hypomorphic mice display disrupted ventral hippocampal–nucleus accumbens transmission [79].

Treatments that induce neurogenic, antidepressant and anxiolytic effects typically do so after chronic - but not subchronic - duration [2], [3], [17], [18], [80], [81]. The present study demonstrates that NRG1 has neurogenic and antidepressant effects with subchronic (72 h) treatment. These effects were measured 28 d after cessation of treatment (and long after exogenous NRG1β would have degraded), a delay that mirrors the maturation period of neurons born during treatment [82]. Given that these newborn neurons are then in a state of increased plasticity [82], our results suggest that boosting the number of highly plastic neurons in the ventral hippocampus can mediate antidepressant effects. Notably, 24 h of treatment was sufficient to increase ventral hippocampal cell proliferation but not to produce antidepressant effects, supporting the hypothesis that the latter are due to the hyperplastic state of immature neurons and not simply an increased number of DG cells. A recent study has suggested that behavior in the FST may be neurogenesis-independent [19], in opposition to previous studies [12], [18]. However, our temporal paradigm shows that the antidepressant effects seen here are concomitant with an increase in adult-born granule cells. Although the current data indicate that the antidepressant effects of NRG1 in particular may be neurogenesis-dependent, they also offer support to the hypothesis that established antidepressants such as SSRIs have a delayed onset of clinical effectiveness (and require chronic treatment) due to the delay required for treatment-induced newborn neurons to mature and integrate into DG circuitry.

NRG1 has attracted widespread interest for its involvement in the etiology of psychiatric conditions. Single nucleotide polymorphisms of the *NRG1* gene have been associated with schizophrenia and bipolar disorder [83]–[91]. Mice hypomorphic for NRG1 or ErbB4 show behavioral abnormalities consistent with existing animal models for schizophrenia, including abnormal prepulse inhibition and enhanced response to cannabinoid and dopaminergic agonists [92]–[94]. Studies of clinical populations have revealed decreased peripheral expression of NRG1β in schizophrenic patients that increased with antipsychotic treatment [30], [31], which is particularly interesting as NRG1β can cross the adult BBB and affect brain activity and behavior [33]–[36]. Schizophrenia has been associated with both abnormal NRG1 type-I signaling in the prefrontal cortex [95]–[97] and decreased hippocampal neurogenesis [98], [99]. In particular, some studies suggest that NRG1 levels are decreased in the brains of depressed and schizophrenic patients [95], [96]. Decreased NRG1 activity may negatively affect hippocampal neurogenesis in schizophrenic patients, contributing to the etiology of this disorder. NRG1 administration could both reverse decreases in hippocampal neurogenesis and ameliorate psychiatric symptoms. The potential utility of NRG1β as a therapeutic that could be delivered peripherally is supported by a recent Phase II clinical study examining NRG1β as a treatment for chronic heart conditions, which revealed that peripheral NRG1β administration does not lead to serious adverse effects in humans [100].

In conclusion, this study is the first to suggest that NRG1β, a peripherally and centrally expressed neurotrophic factor that crosses the BBB, increases ventral hippocampal neurogenesis and modulates mood as a putative antidepressant. It is also the first to show that newborn DG cells express ErbB3 from birth to maturity. That the behavioral effects were not observed acutely but were present four weeks after subchronic treatment cessation in a novel administration paradigm is consistent with the neurogenic theory of antidepressant effects, as this time frame corresponds to the maturation and functional integration of newborn neurons within ventral hippocampal circuitry following treatment-stimulated proliferation. These results also highlight the potential for modulation of brain plasticity and behavior by peripheral neurotrophic factors.

## Supporting Information

Figure S1 **BrdU/ErbB3 colocalization is present for at least 28 d after cell birth.** BrdU-IR cells (**red**) express ErbB3 (**green**), 2 h (**A**), 24 h (**Fig. 5**), 7 d (**B**), and 28 d (**C**) after birth. Scale bars = 20 µm.
(PPT)

Figure S2 **Orthogonal views of BrdU/ErbB3 colocalization.** Orthogonal view of BrdU (**red**) / ErbB3 (**green**) colocalized cells from **Fig. 5** and **Fig. S1**. **A**, 2 h; **B**, 24 h; **C**, 7 d; **D**, 28 d.
(PPT)

Figure S3 **Orthogonal views of cell type colocalization.** **A**, orthogonal view of triple-labeled cell from **Fig. 6A**. **B**, orthogonal view of ErbB3-IR/SOX2-IR/nestin-negative cell from **Fig. 6B**. **Red**, ErbB3; **blue**, SOX2; **green**, nestin.
(PPT)
