# Supplementary figures and images for: Subchronic Peripheral Neuregulin-1 Increases Ventral Hippocampal Neurogenesis and Induces Antidepressant-Like Effects
Source: PLoS One. 2011 Oct 19;6(10):e26610. doi: 10.1371/journal.pone.0026610 (PMC3197569; doi:10.1371/journal.pone.0026610)

## Slide 1
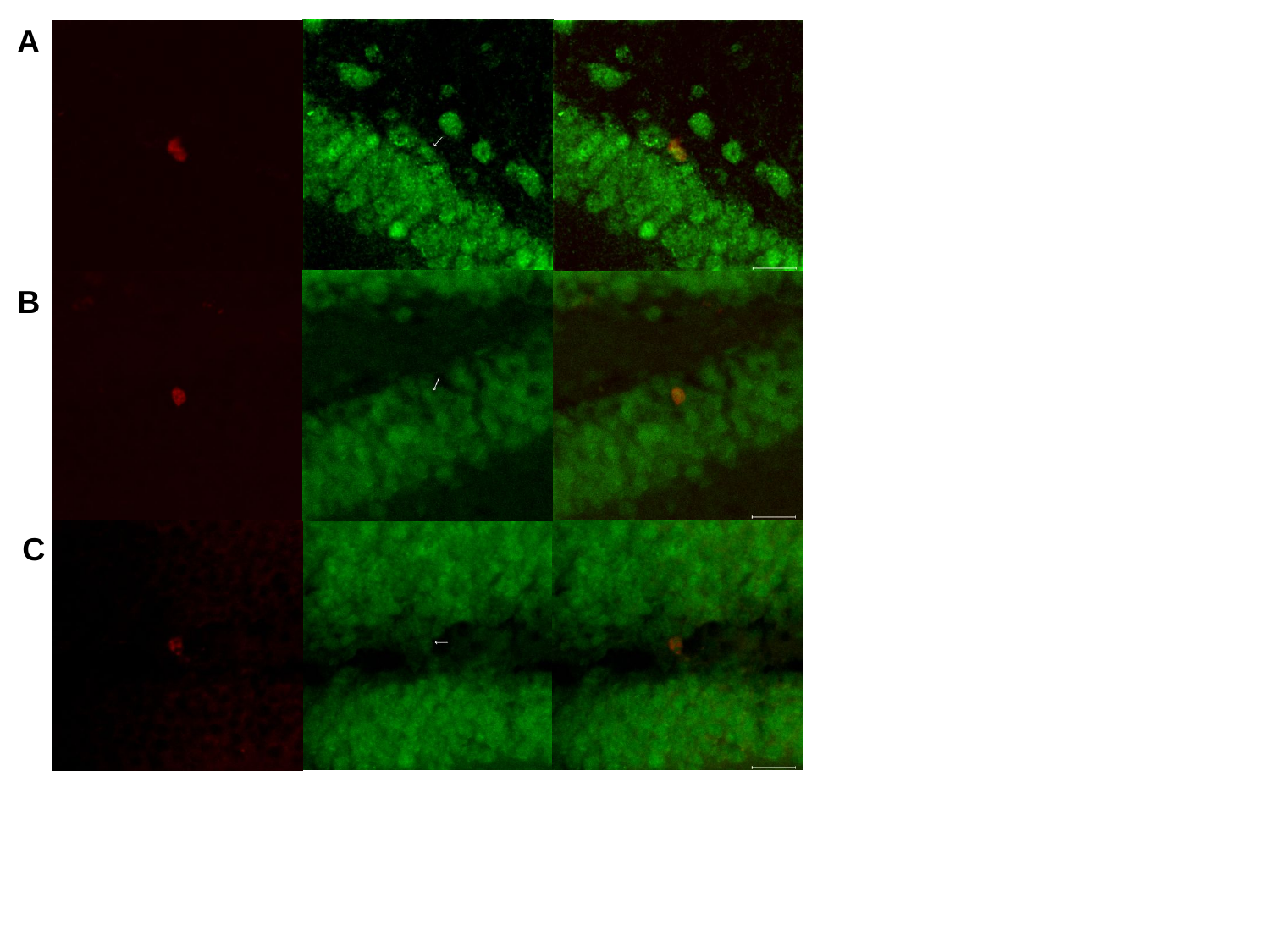

A
B
C

Supplement: Figure S1 — BrdU/ErbB3 colocalization is present for at least 28 d after cell birth. BrdU-IR cells (red) express ErbB3 (green), 2 h (A), 24 h ( Fig. 5 ), 7 d (B), and 28 d (C) after birth. Scale bars = 20 µm. (PPT) [file pone.0026610.s001.ppt]

## Slide 1
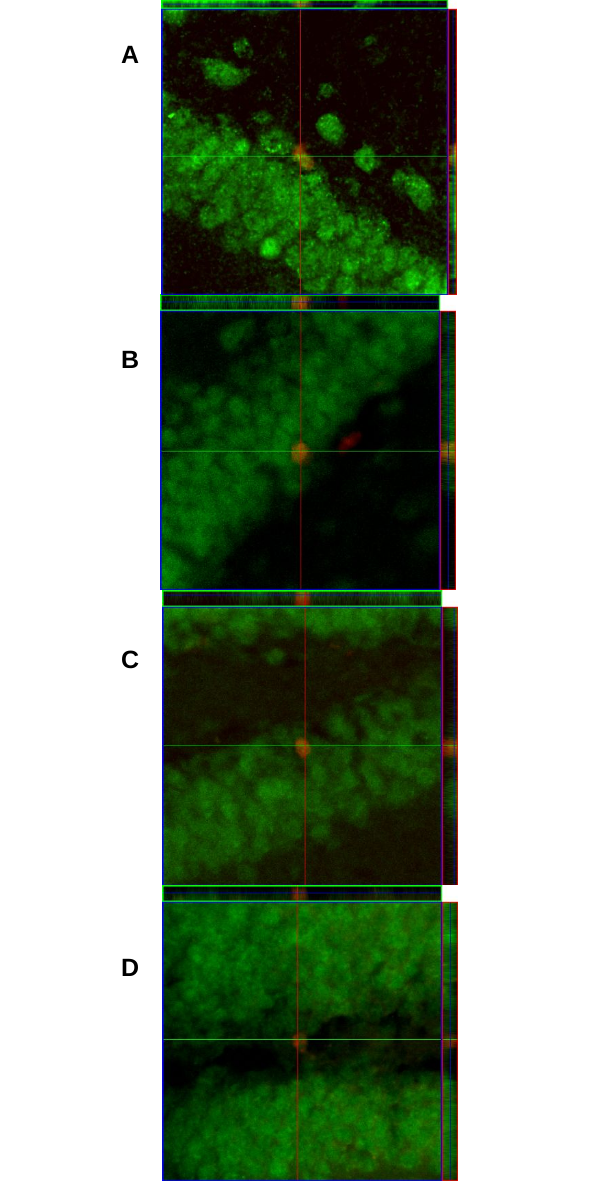

A
B
C
D

Supplement: Figure S2 — Orthogonal views of BrdU/ErbB3 colocalization. Orthogonal view of BrdU (red) / ErbB3 (green) colocalized cells from Fig. 5 and Fig. S1. A, 2 h; B, 24 h; C, 7 d; D, 28 d. (PPT) [file pone.0026610.s002.ppt]

## Slide 1
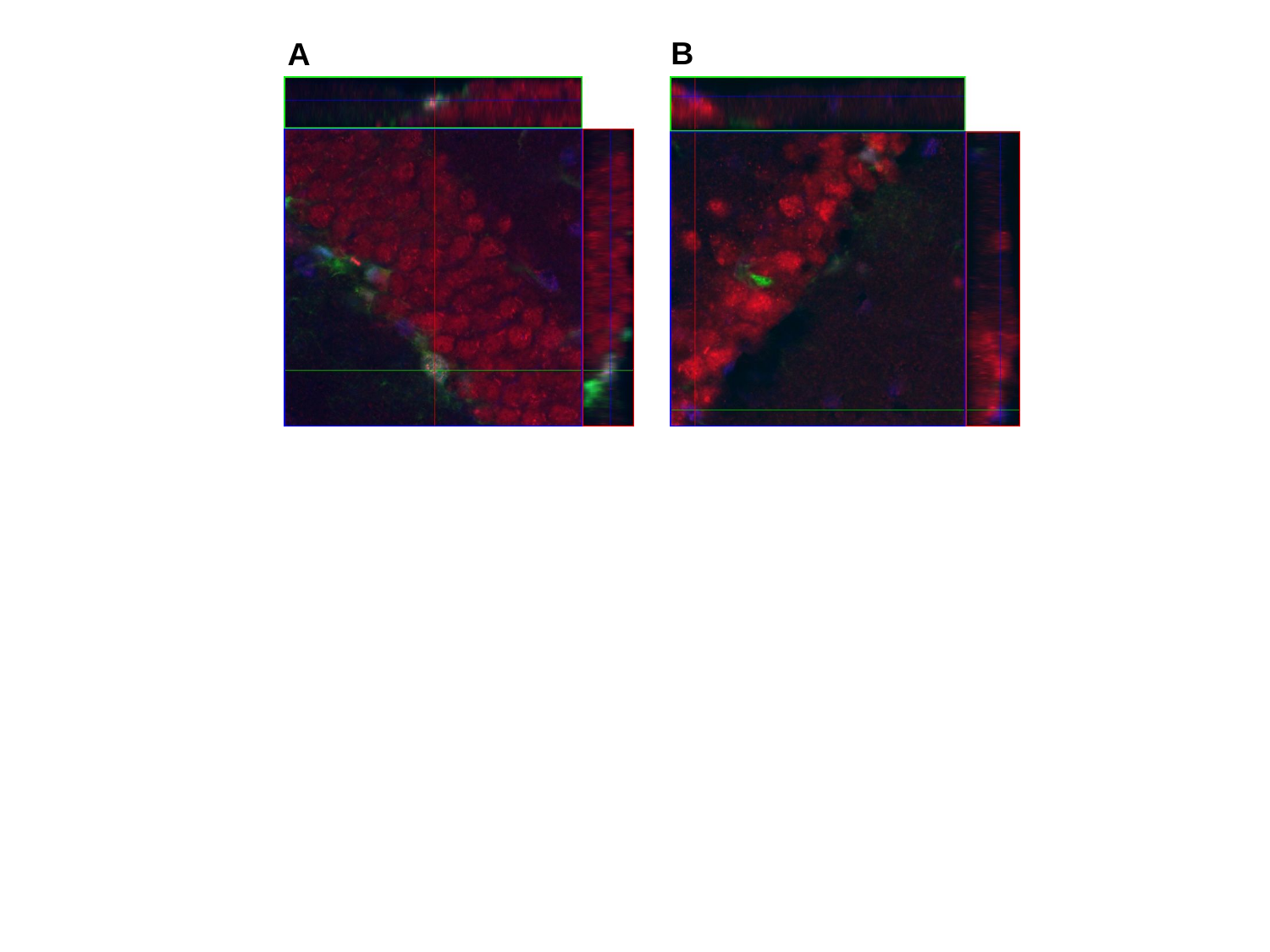

B
A

Supplement: Figure S3 — Orthogonal views of cell type colocalization. A, orthogonal view of triple-labeled cell from Fig. 6A . B, orthogonal view of ErbB3-IR/SOX2-IR/nestin-negative cell from Fig. 6B . Red, ErbB3; blue, SOX2; green, nestin. (PPT) [file pone.0026610.s003.ppt]
